# Three Studies Evaluating the Potential for Lidocaine, Bupivacaine or Procaine to Reduce Pain-Related Behaviors following Ring Castration and/or Tail Docking in Lambs

**DOI:** 10.3390/ani11123583

**Published:** 2021-12-17

**Authors:** Alison Small, Manon Fetiveau, Robin Smith, Ian Colditz

**Affiliations:** 1 CSIRO Agriculture and Food, Chiswick, New England Highway, Armidale, NSW 2358, Australia; manon.feti@outlook.fr (M.F.); ian.colditz@csiro.au (I.C.); 2 Ecole Nationale Supérieure des Sciences Agronomiques de Bordeaux Aquitaine, CEDEX, 33175 Gradignan, France; 3 4c Design, 100 Borron Street, Glasgow G4 9XG, UK; robin@4cdesign.co.uk

**Keywords:** analgesia, local anesthetic, sheep, Lignocaine, Elastrator, husbandry, rubber ring

## Abstract

**Simple Summary:**

Lambs are routinely castrated and/or tail docked. Local anesthesia could improve lamb welfare, but data on the duration of effect of local anesthetics are not available. This study evaluated the efficacy of lidocaine, procaine, or bupivacaine in terms of the behavioral response to castration and/or tail docking. The benefits of local anesthetics were modest. The effects of procaine appear to last longer than lidocaine, while bupivacaine is slower to take effect but may provide longer-lasting pain relief. The duration of action of local anesthetics is limited in sheep, and detailed behavioral evaluations are required in the first hour post-procedure to observe efficacy.

**Abstract:**

The use of local anesthesia at the time of ring castration and tail docking can improve lamb welfare. However, few local anesthetics are registered for sheep, and data on their duration of effect is limited. Three studies were conducted to evaluate the efficacy of procaine (P), lidocaine (L), and bupivacaine (B) in terms of observed alleviation of behavioral responses to castration and/or tail docking in 10-min blocks in the first 60 min post-treatment. In each study, comparisons were made between two groups of lambs castrated and/or tail docked with rubber rings and either receiving the agent using the NUMNUTS^®^ instrument (N) or receiving no anesthetic agent (RR). Acute pain behavior was lower in NL (*n* = 28) than RRL (*n* = 15) males in the first 10 min post-procedure (*p* < 0.05); lower in NB (*n* = 16) than RRB (*n* = 16) males in periods 10–20 min (0.05 < *p* < 0.01), 20–30 min (*p* < 0.05) and 40–50 min (0.05 < *p* < 0.01); lower in NB (*n* = 16) than RRB (*n* = 16) females between 20 and 40 min post-procedure (0.05 < *p* < 0.01); lower in NP (*n* = 8) than RRP (*n* = 7) males in period 10–20 min (0.05 < *p* < 0.01), and lower in NP (*n* = 9) than RRP (*n* = 9) females in periods 0–10 min (0.05 < *p* < 0.01), and 10–40 min (*p* < 0.05). Benefits were modest, and the effects of procaine appear to last longer than lidocaine, while bupivacaine is slower to take effect than either procaine or lidocaine but may provide longer-lasting pain relief. The duration of action of local anesthetics is short in sheep, and detailed behavioral evaluations are required in the first hour post-procedure to establish efficacy.

## 1. Introduction

Castration and tail-docking of lambs are common husbandry practices and are considered to be very painful for the animals [1,2]. Castration prevents mismating and allows males and females to live together [3]. It also reduces aggressive behavior when the males reach sexual maturity and the aversive flavor of meat once animals are killed. Tail-docking reduces the deposit of feces on the tail and, therefore reduces the risk of flystrike [4,5]. Castration and tail docking are often carried out using a rubber ring, and pain relief must be used when lambs are over 6 months of age in Australia [6], or when lambs are over 7 days of age in the UK [7,8]. Local anesthesia could provide some pain relief, but currently, access to these drugs requires a veterinary prescription. In addition, the process of injecting a local anesthetic into the tissues requires a reasonable level of skill to avoid self-injection. Furthermore, the use of multiple syringes and needles on the farm leads to environmental and safety concerns regarding the disposal of consumables and sharps.

The NUMNUTS^®^ instrument allows safe and consistent delivery of local anesthetic as a single central deposit to the tail or scrotum of lambs at the time of rubber ring castration or tail docking [9]. Lidocaine is registered for use in sheep in Australia, but at the time of the study, there were no local anesthetics registered for use in sheep in the UK. Thus, there is a need to evaluate different local anesthetics for their potential to provide pain relief to lambs undergoing ring castration and/or tail docking to inform potential future product registrations. Previous studies suggest that lidocaine provides rapid onset (5–10 min) but short duration (1–2 h) whereas bupivacaine and procaine have a slower onset (10–20 min) but longer duration (4–6 h) [10,11,12,13]. Three studies were conducted to evaluate the efficacy of these three local anesthetics in terms of onset and duration of observed alleviation of behavioral responses in the first hour following ring castration and/or tail docking in lambs, under experimental conditions designed to replicate commercial sheep husbandry conditions. Within each experiment, the null hypothesis tested was that there were no significant differences in the number of acute pain-related behaviors performed by lambs receiving the local anesthetic and lambs that did not receive the local anesthetic.

## 2. Materials and Methods

The animal phase of this study was carried out at the Moredun Research Institute, Midlothian Scotland, under UK Home Office Licence PPL 70/8075. The experiments were reviewed and approved by the Moredun Research Institute Animal Welfare and Ethical Review Body (AWERB), reference E02/17 and E25/18. Two studies were conducted in the spring of 2017 and one in the Spring of 2018 (Table 1). Each experiment was a blinded, randomized block design, randomization being carried out by pre-assigning the order of administration of treatment to each gender, and lambs being processed in the order in which they were captured for treatment. Lamb numbers and gender balance were influenced by lamb supply constraints, while a technical fault resulting in loss of video footage during part of Experiment 1 led to unbalanced numbers of lambs in treatments in that experiment.

The study animals were unweaned twin-born cross-bred lambs with Scottish Greyface or Scottish Mule mothers and Border Leicester fathers, aged between 3 to 5 weeks at the time of the procedure. Bodyweights were not recorded. The lambs with their mothers were transferred to pens bedded with straw one week prior to the experiment. They had water at their disposal and they were fed after the experiments. Pens (6 m × 9 m) were encircled by opaque gates which prevented visual contact or interaction between lambs from different pens.

Treatments were administered in cohorts of 6–8 lambs with each pen containing lambs of mixed gender assigned to a single treatment group. Four cohorts were processed on each study day, two in the morning and two in the afternoon. Immediately prior to the processing of a cohort, lambs were separated from their mothers and held in a small pen adjacent to the observation pen until the individual treatment had been applied. The ewes were released into the observation pen. There were two observation pens, each housing one cohort of lambs and ewes at a time. The observation pens were clearly labeled A or B, and time was visible on a wall-mounted clock in each pen. Observation pens were identical to the home pens, and in the same barn as those to which the animals were acclimated. For all treatments, the operator caught a lamb and restrained it on its back in a lamb marking cradle. For control animals (ring castration and/or tail-docking without anesthetic, RRL, RRB, and RRP), the rubber ring was applied using a standard Elastrator tool (YNR Instruments, Manchester, UK) and lambs were given no anesthetic. This mimics the current industry standard practice. For NL, NB, and NP lambs, the rubber ring was applied and 1.5 mL of the assigned local anesthetic (Table 1) was immediately administered at the site of ring application, using the NUMNUTS^®^ tool (Senesino Ltd., Glasgow, UK). In all lambs, the ring was applied at the third palpable joint from the base of the tail. The operator applying the treatments was a veterinarian with over 30 years of experience using rubber rings for castration and/or tail-docking of lambs and had used the NUMNUTS^®^ device on more than 50 lambs prior to participating in the pilot studies. Prior to treatment application, the device was visually assessed and discharged twice to ensure that it was working properly. Previously, the volume delivered by the device had been checked by discharging the instrument into a small measuring cylinder (data not shown). Following treatment of each lamb, it was released into the observation pen containing its mother.

The local anesthetics evaluated were lidocaine (Ilium Lignocaine 20, Troy Laboratories, Sydney, Australia); bupivacaine (Chirocaine, AbbVie, Inc., North Chicago, IL, USA), and procaine with Adrenaline (Adrenacaine, Norbrook Laboratories, Newry, UK). Lambs were released into the observation pens to join their mothers immediately after treatment.

Video cameras were used to record the behavior of lambs. For each pen, one camera was mounted on roofing rafters at each end of the pen to provide a view of the entire area available for the lambs. The cameras were connected to digital video recorders and footage captured by a video management software (Huawei Technologies Co., Ltd., Reading, UK). Identification for observation of behavior on video records was provided by colored spray numbers/symbols applied to the wool of lambs before treatments. Identification marks were randomized across treatments within a pen. The assessment of the behavior post-treatment was carried out off-line, after all the studies had been completed, by an operator blinded to treatment and active. This operator was trained by an experienced animal behavior assessor, and a randomized subset (10%) of lambs were separately assessed by the trainer to ensure concurrence between operators (96% concurrence, data not presented). For each lamb, the video footage was assessed continuously for the first hour after treatment. The number of times the lamb expressed active pain avoidance behaviors in every 10-min block after the lamb had been returned to the pen was counted, until 60 min had elapsed. The active pain behaviors counted were according to a pre-determined ethogram adapted from Paull et al. 2012 [14] (Table 2).

Data were analyzed using R statistical software [15]. Initially, a repeated measures mixed model was used, fitting lamb ID as a random effect and treatment, time period, and gender as fixed effects, including first-order interactions where significant. Gender by time period interactions were significant, so male and female data were analyzed separately. Data could not be transformed to satisfy a normal distribution so were analyzed within time points and as the entire 60-min period using Welch’s *t*-test. Total pain avoidance behaviors; restlessness and foot stamping/kicking were analyzed separately, whereas counts of rolling; jumping; licking at the wound site and easing quarters were too low to permit separate analysis. *p* < 0.05 was used to indicate statistical significance, while 0.1 > *p* > 0.05 was considered to indicate a tendency towards significance.

## 3. Results

**Figure 1:**
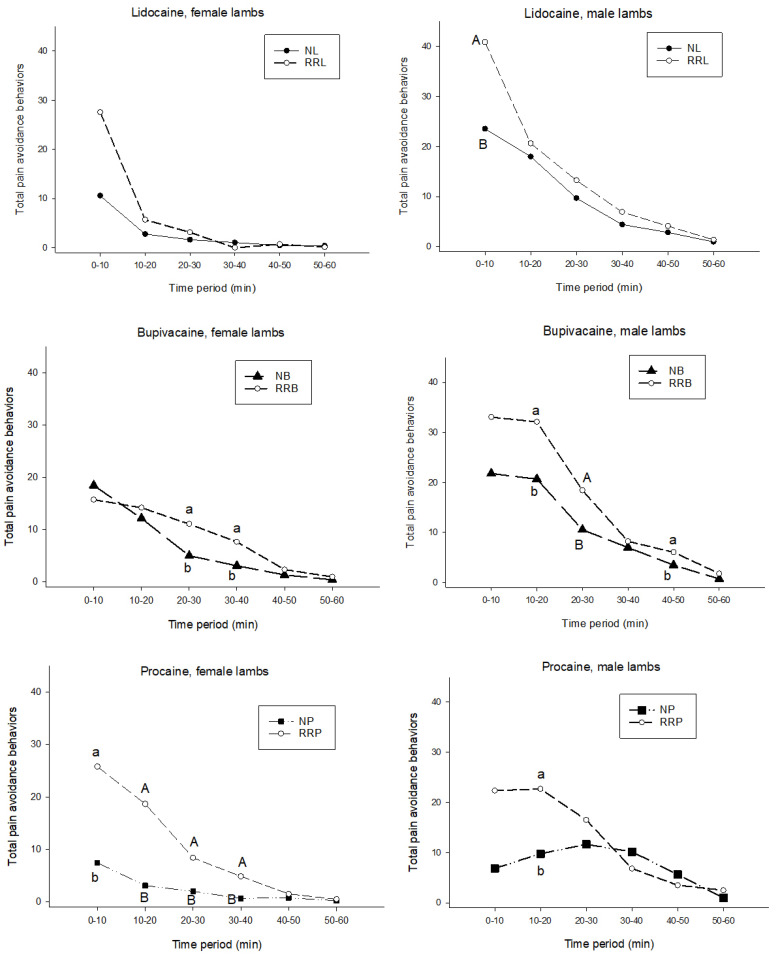
Total acute pain avoidance behaviors in ten-min blocks post ring castration and/or tail docking in female (**left**) and male (**right**) lambs receiving lidocaine (**top**); bupivacaine (**middle**) or procaine (**bottom**) as compared with lambs receiving no local anesthetic. Within each experiment, means within a time period with differing uppercase superscripts (A, B) differ significantly (*p* < 0.05), while differing lowercase superscripts (a, b) indicate a tendency to differ (0.1 > *p* > 0.05). Error bars not shown to optimize visual clarity of charts (see Table 3 and Table 4 for standard deviations). RRL ring only, no local anesthetic; NL ring applied with lidocaine administered using the Numnuts^®^ tool; RRB ring only, no local anesthetic; NB ring applied with bupivacaine administered using the Numnuts^®^ tool; RRP ring only, no local anesthetic; NP ring applied with procaine administered using the Numnuts^®^ tool.

All local anesthetic agents provided some mitigation of total acute pain avoidance behaviors (Figure 1). For lidocaine, this mitigation was only evident in the first ten-min block post castration and/or tail docking, and was statistically significant (*p* < 0.05) in male lambs (23.55 ± 20.43 versus 40.87 ± 27.44, Table 3), but not in female lambs (10.58 ± 12.46 versus 27.57 ± 23.64, Table 4). In male lambs provided with lidocaine, there was a tendency for reduced counts of acute pain avoidance behaviors over the entire 60-min period (59.49 ± 34.19 versus 87.27 ± 51.49, Table 3). In male lambs provided with lidocaine, there was a significant reduction in restlessness in the 30–40 min block (3.87 ± 1.92 versus 2.28 ± 2.86, Table 5), and a tendency for a reduction in restlessness (31.28 ± 14.42 versus 41.47 ± 17.32) and in foot-stamping and kicking (21.76 ± 19.21 versus 38.27 ± 31.00, Table 6) over the entire 60-min period. In female lambs provided with lidocaine, there was a statistically significant increase in acute pain-related behaviors and restlessness in the 30–40 min block (0 ± 0 versus 1.05 ± 1.81, Table 4 and 0 ± 0 versus 0.89 ± 1.59, Table 7, respectively).

In male lambs provided with bupivacaine, there was a significant reduction in acute pain avoidance behaviors in the 20–30 min block (18.44 ± 10.50 versus 10.56 ± 5.60), a tendency for reduction in the 10–20 and 40–50 min blocks (32.13 ± 20.87 versus 20.69 ± 11.45 and 6.06 ± 4.58 versus 3.44 ± 2.63 respectively, Table 3), and a significant reduction in counts of acute pain avoidance behaviors over the entire 60-min period (64.19 ± 27.72 versus 99.69 ± 53.60). For restlessness, there was a significant reduction in counts in the 20–30 min block (10.44 ± 5.06 versus 6.69 ± 3.22) and in the 40–50 min block (5.81 ± 4.21 versus 2.94 ± 2.08), and a tendency for a reduction in restlessness over the entire 60-min period (60.06 ± 31.94 versus 43.19 ± 12.97, Table 5).

In female lambs provided with bupivacaine, there were no significant differences in total acute pain avoidance behaviors to those provided no local anesthetic for the first 20 min post-tail-docking, but in the 20–30 min and the 30–40 min blocks there was a tendency (0.1 > *p* > 0.05) for a reduction in total acute pain avoidance behaviors in those receiving bupivacaine (5.00 ± 4.30 and 3.08 ± 3.12 versus 11.07 ± 10.43 and 7.67 ± 9.04 respectively, Table 4). There were no significant differences between groups at the 40–50 min and 50–60 min blocks. This pattern of no significant differences in the first 20 min, a tendency to differ in the 20–30 min and the 30–40 min blocks, and no significant differences in the 40–50 min and 50–60 min blocks was mirrored in counts of restlessness (Table 7).

In male lambs receiving procaine, there was a significant reduction in restlessness during the 10–20 min block (12.67 ± 5.47 versus 5.36 ± 4.98), which contributed to a tendency for reduced total acute pain avoidance behaviors in that time period (22.67 ± 13.23 versus 9.75 ± 8.58). Significant (*p* < 0.05) differences in restlessness were observed between male lambs receiving lidocaine (12.86 ± 7.98) and those receiving no local anesthetic (18.87 ± 7.39) in that first 10-min block (Table 5), and a tendency (0.1 > *p* > 0.05) for foot stamping or kicking to be less in lidocaine-treated lambs (9.03 ± 10.82) than in those receiving no local anesthetic (18.67 ± 17.65, Table 6). There were no significant differences between the lidocaine-treated group and the group receiving no local anesthetic in female lambs in terms of foot-stamping or kicking (Table 8).

In female lambs provided with procaine, there were significant reductions in acute pain-related behaviors in the 10–20 min (18.63 ± 12.30 versus 3.11 ± 4.04), 20–30 min (8.38 ± 5.86 versus 2.00 ± 2.55), and 30–40 min (4.88 ± 3.40 versus 0.67 ± 1.00) blocks, and a tendency for a reduction in the 0–10 min block (25.75 ± 18.37 versus 7.44 ± 18.03). There were significant reductions in restlessness in the 10–20 min block (12.67 ± 5,47 versus 5.63 ± 4.98), and in foot-stamping and kicking in the 10–20 min (8.88 ± 5.74 versus 2.44 ± 3.54), 20–30 min (7.00 ± 5.01 versus 1.33 ± 1.94) and 30–40 min (4.75 ± 3.37 versus 0.56 ± 1.01) blocks in female lambs receiving procaine. Over the entire 60-min period, there was a significant reduction in acute pain-related behaviors (14.22 ± 23.58 versus 59.63 ± 28.35, Table 4), restlessness (9.56 ± 15.76 versus 31.13 ± 14.89, Table 7), and foot stamping and kicking (2.22 ± 4.55 versus 10.38 ± 7.29, Table 8).

## 4. Discussion

Application of a rubber ring to the scrotum and/or tail was accompanied by the expression of acute pain avoidance behaviors, restlessness, and foot stamping or kick, all of which had declined to a low level by 60 min. As expected, local anesthesia resulted in reductions in pain-related behaviors but did not eliminate the behavioral response. The period of influence and duration of effect on behaviors differed between local anesthetics. The duration of the significant effect of lidocaine was very short, significant effects being recorded only in the first 10 min post-procedure, and in male lambs only. This is a substantially shorter period than that reported previously: e.g., by Mellema et al. (2006) [16], who observed reductions in pain-related behavior in the first 2 h post-procedure; Stewart et al. (2014) [17] who reported reductions in abnormal lying, activity, and postures in the 3 h post-procedure; Thornton and Waterman-Pearson (1999) [18], who reported abolition of behavioral responses and scrotal nociception in the first 8 h post-procedure; and Kent et al. (1998) [19], who reported significant reductions in active pain behavior and time spent in abnormal postures over 3 h post-procedure. Key differences between all these studies and the current study are the dose of lidocaine delivered and the injection pattern. Mellema et al. delivered 4 mg/kg, distributed into the scrotal neck and spermatic cords; Stewart et al. delivered 120 mg, distributed into the testes and scrotal neck, and Thornton and Waterman-Pearson delivered 60 mg, distributed into the spermatic cords, testes, and scrotal neck. In the current study, 30 mg was delivered as a central injection into the scrotal neck immediately after the ring was applied, which is more similar to the methodology used by Kent et al., who delivered 4 mg into each side of the scrotal neck using a needleless injector, immediately after the ring was applied. Differences between that study and the current study include the age of lamb (5–8 days as compared to 3–5 weeks in the current study), and the formulation of lidocaine used (Kent et al. used lidocaine with adrenaline, the current study lidocaine alone). Although many pre-castration injection protocols include the testes and/or the spermatic cords as well as the scrotal neck, injection of lidocaine into the scrotal neck alone has been shown to markedly reduce or abolish the cortisol response to ring castration [19,20,21]. The data presented here also differ from the findings of Small et al. (2020) [9], who observed reductions in acute pain avoidance behaviors during the first 20 min (males) and first 35 min (females) in ring marked lambs receiving a single bolus of lidocaine at the site of ring application, also using the Numnuts^®^ instrument. That study differs from the current study in that it was a field-based study, so the behavioral repertoire of lambs likely differed as compared to the pen-based context of the current study.

The onset of anesthetic effect of bupivacaine was delayed in the current study in comparison with lidocaine and procaine, with reductions in pain-related behaviors not being observed until the 10–20 min observation block. Although there is published literature pertaining to the use of bupivacaine for epidural or nerve block administration in sheep indicating a delay in onset of anesthesia for 10–45 min, and duration of 3 to 9 h [10,22,23], there appears to be little published data on the use of bupivacaine for castration and/or tail docking. Graham et al. (1997) [24] delivered 1.25 mg of bupivacaine subcutaneously at the site of ring application, prior to ring application, to 3-week-old lambs undergoing ringtail docking and observed significant reductions in pain-related behavior and abnormal postures in the 3 h post-procedure, and a significant reduction in cortisol response. Use of the same dose as an epidural injection was less effective than subcutaneous administration. There is a similar dearth of information on procaine as an anesthetic for castration and/or tail docking in lambs. Molony et al. (2012) [25] administered 15 mg of procaine with adrenaline to the spermatic cords of 2–3-day-old lambs, immediately after rubber ring application, using a needleless injector. They observed a significant reduction in active pain behaviors and abnormal lying postures in the first hour post-procedure. Our data align somewhat with this finding: we observed significant reductions in active pain behaviors in the first 40 min post ringtail docking, but only a trend for reduction in active pain behaviors in the first 20 min following castration.

Although each agent was assessed separately in the current study, it is evident that there are marked differences between the agents in terms of onset and duration of action. This is likely to be due to either difference in the pharmacokinetics of the agents: for example, the elimination half-lives of lidocaine (17–62 min [26,27]) and bupivacaine (118–142, dependent on route of administration [28,29]) are markedly different; or to differences in both dose provided (a fixed volume of 1.5 mL of each agent being used) and minimum effective dose in sheep. The inclusion of adrenaline in the procaine formulation used in the study could be a further source of variation between agents in pharmacodynamics [27,28,30]. Unfortunately, a commercial supply of procaine without adrenaline could not be found. In terms of optimizing local anesthesia for lamb castration, further work on determining the optimal dose of any one agent with and without adrenaline is important, while the development of a combination formulation of a rapid-onset agent with a prolonged-duration agent (e.g., procaine + bupivacaine) may be of value. Lizarraga et al. (2013) [11] found that a combination of lidocaine with bupivacaine, when used in a metacarpal block, provided no benefit over bupivacaine alone, but the direct inference of those findings to use for tail-docking and/or castration may not be appropriate, as clearance of the agents will differ depending on the local conditions (e.g., vascularity) of the tissues [27].

The NUMNUTS^®^ tool has been designed to allow administration of a fixed volume (1.5 mL) of local anesthetic at the time of ring application, thereby reducing the number of operations from two to one, to optimize workflow under commercial lamb marking conditions. Thus, there is not the opportunity to afford the lamb a period of time for the local anesthetic to take effect prior to ring application. Ideally, the agent administered using the NUMNUTS^®^ tool would have an extremely rapid onset of effect to maximize the animal welfare benefits.

## 5. Conclusions

These evaluations indicate that the anesthetic benefits to lambs undergoing ring castration and/or tail docking of individual local anesthetics differ. Benefits of procaine (with adrenaline) appear to be more sustained than those of lidocaine, with a similar early onset of effect, while the benefits of bupivacaine are realized later than lidocaine or procaine, and may be sustained for a greater period of time than lidocaine. A larger, controlled simultaneous comparison of the agents is required to confirm these findings. Further research should take into consideration the fact that the duration of action of local anesthetics is limited in sheep, and detailed behavioral evaluations are required in the first hour post-procedure. Evaluations after that first hour or thereafter are not pharmacologically relevant and thus are unlikely to show any effect of the local anesthetic agent.

In terms of animal welfare, further work on determining the optimal dose of any one agent is important, while the development of a combination formulation of procaine and bupivacaine may accrue the benefits of both rapid onset and sustained pain relief.

## Figures and Tables

**Table 1:** Distribution of lambs by husbandry procedure and treatment in the three trials.

Code	Procedure	Therapeutic Agent	No. Lambs
*Experiment 1–Evaluation of Lidocaine 4–6 April 2017*			
RRL	Males: Ring castration and tail-dock Females: Ring tail-dock	No anesthetic Control	15 males and 8 females
NL	Males: Ring castration and tail-dock Females: Ring tail-dock	Lidocaine 2% (1.5 mL/site) 30 mg per site	28 males and 28 females
*Experiment 2–Evaluation of Bupivacaine 25–26 April 2017*			
RRB	Males: Ring castration and tail-dock Females: Ring tail-dock	No anesthetic Control	16 males and 16 females
NB	Males: Ring castration and tail-dock Females: Ring tail-dock	Levobupivacaine 7.5 mg/mL (1.5 mL/site) 10.75 mg per site	16 males and 16 females
*Experiment 3–Evaluation of Procaine 10–11 May 2018*			
RRP	Males: Ring castration Females: Ring tail-dock	No anesthetic Control	7 males and 9 females
NP	Males: Ring castration Females: Ring tail-dock	Procaine 5% + Adrenaline (1.5 mL/site) 75 mg per site	8 males and 9 females

**Table 2:** Active pain avoidance behaviors were assessed during observation of recordings of animals described in Table 1 (adapted from Paull et al. 2012 [14]). In every 10-min block after treatment, the number of occasions each behavior was displayed lamb was counted.

Behavior	Abbreviation	Description
Restlessness	RST	The number of times lamb stood up and laid down. Instances of lamb rising as far as its knees included in the one count.
Kicking/foot-stamping	FSK	Either a front or hind limb (usually hind limb) was lifted and forcefully placed on the ground while standing or was used to kick while standing or lying.
Rolling	RL	Lamb rolled from lying on one side to the other without getting up. Half rolls where the lamb rolled on its back and then returned to lying on the same side included.
Jumping	JMP	Lamb moved forward using bunny hops with its hind limbs
Licking/biting wound site	LBW	Movement of the head beyond the shoulder, including both looking and touching at the source of pain and grooming.
Easing quarters	EQ	Abnormally lowers rear quarters (standing) or attempts to keep quarters off the ground (lying).
Total acute pain avoidance behaviors	RST + FSK + RL + JMP + LBW + EQ	All pain avoidance behaviors pooled.

**Table 3:** Mean (± standard deviation) count of total active pain-related behaviors expressed by male lambs. Within each experiment, means within a column with differing uppercase superscripts (A, B) differ significantly (*p* < 0.05), while differing lowercase superscripts (a, b) indicate a tendency to differ (0.1 > *p* > 0.05). RRL ring only, no local anesthetic; NL ring applied with lidocaine administered using the Numnuts^®^ tool; RRB ring only, no local anesthetic; NB ring applied with bupivacaine administered using the Numnuts^®^ tool; RRP ring only, no local anesthetic; NP ring applied with procaine administered using the Numnuts^®^ tool.

Treatment	0–10 min	10–20 min	20–30 min	30–40 min	40–50 min	50–60 min	0–60 min
*Experiment 1—Evaluation of lidocaine 4–6 April 2017*							
RRL (*n* = 15)	**40.87 ^A^** (±27.44)	20.97 (±12.61)	13.27 (±10.01)	6.93 (±4.48)	4.13 (±4.23)	1.40 (±2.10)	87.27 ^a^ (±51.48)
NL (*n* = 28)	**23.55 ^B^** (±20.43)	18.00 (±12.24)	9.69 (±5.66)	4.41 (±5.77)	2.86 (±3.30)	0.97 (±1.70)	59.48 ^b^ (±34.19)
*Experiment 2—Evaluation of bupivacaine 25–26 April 2017*							
RRB (*n* = 16)	33.06 (±30.33)	32.13 ^a^ (±20.87)	**18.44 ^A^** (±10.50)	8.25 (±7.51)	6.06 ^a^ (±4.58)	1.75 (±2.74)	**99.69 ^A^** (±53.60)
NB (*n* = 16)	21.81 (±10.30)	20.69 ^b^ (±11.45)	**10.56 ^B^** (±5.60)	7.00 (±3.54)	3.44 ^b^ (±2.63)	0.69 (±1.01)	**64.19 ^B^** (±27.72)
*Experiment 3—Evaluation of procaine 10–11 May 2018*							
RRP (*n* = 7)	22.33 (±21.24)	22.67 ^a^ (±13.23)	16.50 (±12.01)	6.83 (±4.07)	3.50 (±2.66)	2.50 (±3.15)	74.33 (±46.75)
NP (*n* = 8)	6.88 (±4.82)	9.75 ^b^ (±8.58)	11.63 (±7.61)	10.13 (±8.82)	5.63 (±4.34)	1.00 (±1.41)	45.00 (±28.96)

**Table 4:** Mean (± standard deviation) count of total active pain-related behaviors expressed by female lambs. Within each experiment, means within a column with differing uppercase superscripts (A, B) differ significantly (*p* < 0.05), while differing lowercase superscripts (a, b) indicate a tendency to differ (0.1 > *p* > 0.05). RRL ring only, no local anesthetic; NL ring applied with lidocaine administered using the Numnuts^®^ tool; RRB ring only, no local anesthetic; NB ring applied with bupivacaine administered using the Numnuts^®^ tool; RRP ring only, no local anesthetic; NP ring applied with procaine administered using the Numnuts^®^ tool.

Treatment	0–10 min	10–20 min	20–30 min	30–40 min	40–50 min	50–60 min	0–60 min
*Experiment 1—Evaluation of lidocaine 4–6 April 2017*							
RRL (*n* = 8)	27.57 (±23.64)	5.71 (±3.99)	3.14 (±4.02)	**0 ^A^** (0)	0.71 (±0.76)	0.14 (±0.38)	37.29 (±31.52)
NL (*n* = 28)	10.58 (±12.46)	2.79 (±4.17)	1.63 (±2.65)	**1.05 ^B^** (±1.81)	0.47 (±0.84)	0.42 (±0.84)	16.95 (±18.20)
*Experiment 2—Evaluation of bupivacaine 25–26 April 2017*							
RRB (*n* = 16)	15.73 (±15.00)	14.20 (±12.08)	11.07 ^a^ (±10.43)	7.67 ^a^ (±9.04)	2.33 (±3.51)	0.93 (±1.33)	51.93 (±44.70)
NB (*n* = 16)	18.46 (±21.07)	12.15 (±12.34)	5.00 ^b^ (±4.30)	3.08 ^b^ (±3.12)	1.31 (±1.93)	0.38 (±1.93)	40.38 (±35.67)
*Experiment 3—Evaluation of procaine 10–11 May 2018*							
RRP (*n* = 9)	25.75 ^a^ (±18.37)	**18.63 ^A^** (±12.30)	**8.38 ^A^** (±5.68)	**4.88 ^A^** (±3.40)	1.50 (±2.14)	0.50 (±1.07)	**59.63 ^A^** (±28.35)
NP (*n* = 9)	7.44 ^b^ (±18.03)	**3.11 ^B^** (±4.04)	**2.00 ^B^** (±2.55)	**0.67 ^B^** (±1.00)	0.78 (±1.09)	0.22 (±0.67)	**14.22 ^B^** (±23.58)

**Table 5:** Mean (± standard deviation) count of restlessness expressed by male lambs. Within each experiment, means within a column with differing uppercase superscripts (A, B) differ significantly (*p* < 0.05), while differing lowercase superscripts (a, b) indicate a tendency to differ (0.1 > *p* > 0.05). RRL ring only, no local anesthetic; NL ring applied with lidocaine administered using the Numnuts^®^ tool; RRB ring only, no local anesthetic; NB ring applied with bupivacaine administered using the Numnuts^®^ tool; RRP ring only, no local anesthetic; NP ring applied with procaine administered using the Numnuts^®^ tool.

Treatment	0–10 min	10–20 min	20–30 min	30–40 min	40–50 min	50–60 min	0–60 min
*Experiment 1—Evaluation of lidocaine 4–6 April 2017*							
RRL (*n* = 15)	**18.87 ^A^** (±7.39)	9.00 (±4.86)	6.40 (±4.39)	**3.87 ^A^** (±1.92)	2.53 (±1.85)	0.80 (±1.47)	41.47 ^a^ (±17.32)
NL (*n* = 28)	**12.86 ^B^** (±7.98)	8.66 (±4.24)	5.07 (±2.42)	**2.28 ^B^** (±2.86)	1.86 (±2.35)	0.55 (±1.12)	31.28 ^b^ (±14.42)
*Experiment 2—Evaluation of bupivacaine 25–26 April 2017*							
RRB (*n* = 16)	17.69 (±12.78)	17.75 (±16.61)	**10.44 ^A^** (±5.06)	6.81 (±5.91)	**5.81 ^A^** (±4.21)	1.56 (±2.73)	60.06 ^a^ (±31.94)
NB (*n* = 16)	16.50 (±6.00)	11.38 (±3.88)	**6.69 ^B^** (±3.22)	5.31 (±3.02)	**2.94 ^B^** (±2.08)	0.38 (±0.72)	43.19 ^b^ (±12.97)
*Experiment 3—Evaluation of procaine 10–11 May 2018*							
RRP (*n* = 7)	12.33 (±11.89)	**12.67 ^A^** (±5.47)	8.50 (±5.65)	4.17 (±2.86)	1.67 (±1.51)	1.00 (±1.10)	40.33 (±23.75)
NP (*n* = 8)	4.00 (±3.51)	**5.63 ^B^** (±4.98)	6.38 (±4.78)	4.50 (±3.70)	3.38 (±2.83)	0.50 (±0.76)	24.38 (±17.17)

**Table 6:** Mean (± standard deviation) count of foot-stamping or kicking expressed by male lambs. Within each experiment, means within a column with differing uppercase superscripts (A, B) differ significantly (*p* < 0.05), while differing lowercase superscripts (a, b) indicate a tendency to differ (0.1 > *p* > 0.05). RRL ring only, no local anesthetic; NL ring applied with lidocaine administered using the Numnuts^®^ tool; RRB ring only, no local anesthetic; NB ring applied with bupivacaine administered using the Numnuts^®^ tool; RRP ring only, no local anesthetic; NP ring applied with procaine administered using the Numnuts^®^ tool.

Treatment	0–10 min	10–20 min	20–30 min	30–40 min	40–50 min	50–60 min	0–60 min
*Experiment 1—Evaluation of lidocaine 4–6 April 2017*							
RRL (*n* = 15)	18.67 ^a^ (±17.65)	9.80 (±7.87)	5.60 (±6.15)	2.60 (±3.07)	1.20 (±2.04)	0.40 (±0.83)	38.27 ^a^ (±31.00)
NL (*n* = 28)	9.03 ^b^ (±10.82)	7.17 (±7.64)	3.31 (±3.52)	1.41 (±2.49)	0.59 (±0.91)	0.24 (±0.69)	21.76 ^b^ (±19.21)
*Experiment 2—Evaluation of bupivacaine 25–26 April 2017*							
RRB (*n* = 16)	8.56 (±8.80)	8.06 (±6.94)	4.75 (±4.61)	1.13 (±1.67)	0.25 (±0.68)	0 (0)	22.75 (±16.18)
NB (*n* = 16)	4.44 (±5.81)	10.94 (±22.18)	3.13 (±3.36)	1.06 (±1.65)	0.50 (±1.32)	0.13 (±0.50)	20.19 (±27.01)
*Experiment 3—Evaluation of procaine 10–11 May 2018*							
RRP (*n* = 7)	9.67 (±9.61)	6.83 (±5.04)	6.33 (±7.00)	2.00 (±2.00)	1.33 (±1.97)	1.00 (±1.67)	27.17 (±21.52)
NP (*n* = 8)	2.50 (±2.14)	3.50 (±3.34)	4.25 (±2.96)	3.25 (±4.46)	1.50 (±2.39)	0.25 (±0.71)	15.25 (±11.71)

**Table 7:** Mean (± standard deviation) count of restlessness expressed by female lambs. Within each experiment, means within a column with differing uppercase superscripts (A, B) differ significantly (*p* < 0.05), while differing lowercase superscripts (a, b) indicate a tendency to differ (0.1 > *p* > 0.05). RRL ring only, no local anesthetic; NL ring applied with lidocaine administered using the Numnuts^®^ tool; RRB ring only, no local anesthetic; NB ring applied with bupivacaine administered using the Numnuts^®^ tool; RRP ring only, no local anesthetic; NP ring applied with procaine administered using the Numnuts^®^ tool.

Treatment	0–10 min	10–20 min	20–30 min	30–40 min	40–50 min	50–60 min	0–60 min
*Experiment 1—Evaluation of lidocaine 4–6 April 2017*							
RRL (*n* = 8)	15.29 (±12.37)	4.57 (±3.46)	2.00 (2.24)	**0 ^A^** (0)	0.57 (±0.53)	0.14 (±0.38)	14.64 (±14.20)
NL (*n* = 28)	6.74 (±8.89)	2.21 (±3.57)	1.37 (±2.29)	**0.89 ^B^** (±1.59)	0.37 (±0.76)	0.16 (±0.37)	11.74 (±13.73)
*Experiment 2—Evaluation of bupivacaine 25–26 April 2017*							
RRB (*n* = 16)	11.60 (±10.99)	10.33 (±8.43)	9.60 ^a^ (±9.09)	7.60 ^a^ (±9.16)	2.27 (±3.53)	0.93 (±3.53)	42.33 (±37.06)
NB (*n* = 16)	11.15 (±13.90)	7.85 (±5.29)	4.85 ^b^ (±4.30)	3.00 ^b^ (±3.03)	1.31 (±1.93)	0.31 (±1.93)	28.46 (±22.52)
*Experiment 3—Evaluation of procaine 10–11 May 2018*							
RRP (*n* = 9)	8.50 (±7.21)	**8.88 ^A^** (±5.74)	**7.00 ^A^** (±5.01)	**4.75 ^A^** (±3.37)	1.50 (±2.14)	0.50 (±1.07)	**31.13 ^A^** (±14.89)
NP (*n* = 9)	4.78 (±11.10)	**2.44 ^B^** (±3.54)	**1.33 ^B^** (±1.94)	**0.56 ^B^** (±1.01)	0.33 (±0.71)	0.11 (±0.33)	**9.56 ^B^** (±15.76)

**Table 8:** Mean (± standard deviation) count of foot-stamping or kicking expressed by female lambs. Within each experiment, means within a column with differing uppercase superscripts (A, B) differ significantly (*p* < 0.05), while differing lowercase superscripts (a, b) indicate a tendency to differ (0.1 > *p* > 0.05). RRL ring only, no local anesthetic; NL ring applied with lidocaine administered using the Numnuts^®^ tool; RRB ring only, no local anesthetic; NB ring applied with bupivacaine administered using the Numnuts^®^ tool; RRP ring only, no local anesthetic; NP ring applied with procaine administered using the Numnuts^®^ tool.

Treatment	0–10 min	10–20 min	20–30 min	30–40 min	40–50 min	50–60 min	0–60 min
*Experiment 1—Evaluation of lidocaine 4–6 April 2017*							
RRL (*n* = 8)	9.86 (±12.55)	1.14 (±1.68)	0.86 (±2.27)	0 (0)	0 (0)	0 (0)	6.33 (±10.87)
NL (*n* = 28)	2.68 (±4.45)	0.21 (±0.71)	0.11 (±0.32)	0 (0)	0 (0)	0 (0)	3.00 (±4.92)
*Experiment 2—Evaluation of bupivacaine 25–26 April 2017*							
RRB (*n* = 16)	2.13 (±2.72)	1.33 (±2.23)	0.20 (±0.41)	0 (0)	0 (0)	0 (0)	3.67 (±4.47)
NB (*n* = 16)	3.31 (±4.59)	1.31 (±2.78)	0.08 (±0.28)	0 (0)	0 (0)	0 (0)	4.69 (±6.60)
*Experiment 3—Evaluation of procaine 10–11 May 2018*							
RRP (*n* = 9)	4.50 (±5.61)	**5.00 ^A^** (±5.32)	0.75 (±0.89)	0.13 (±0.35)	0 (0)	0 (0)	**10.38 ^A^** (±7.29)
NP (*n* = 9)	1.44 (±3.97)	**0.33 ^B^** (±0.71)	0.33 (±0.71)	0 (0)	0.11 (±0.33)	0 (0)	**2.22 ^B^** (±4.55)

## Data Availability

Not applicable.
